# Fracture Resistance of Zirconia-Reinforced Lithium Silicate Ceramic Crowns Cemented with Conventional or Adhesive Systems: An In Vitro Study

**DOI:** 10.3390/ma13092012

**Published:** 2020-04-25

**Authors:** Gianmaria D’Addazio, Manlio Santilli, Marco Lorenzo Rollo, Paolo Cardelli, Imena Rexhepi, Giovanna Murmura, Nadin Al-Haj Husain, Bruna Sinjari, Tonino Traini, Mutlu Özcan, Sergio Caputi

**Affiliations:** 1 Department of Medical, Oral and Biotechnological Sciences, University “G. d’Annunzio” of Chieti-Pescara, 66100 Chieti, Italy; gianmariad@gmail.com (G.D.); santilliman@gmail.com (M.S.); mlrollo90@gmail.com (M.L.R.); paolocardelli@hotmail.com (P.C.); imena.rexhepi@gmail.com (I.R.); giovanna.murmura@unich.it (G.M.); scaputi@unich.it (S.C.); 2 Department of Reconstructive Dentistry and Gerodontology, School of Dental Medicine, University of Bern, 3012 Bern, Switzerland; nalhaj88@gmail.com; 3 Division of Dental Biomaterials, Center for Dental and Oral Medicine, Clinic for Reconstructive Dentistry, University of Zurich, 8006 Zurich, Switzerland; mutluozcan@hotmail.com

**Keywords:** adhesive dentistry, prosthetic dentistry, in vitro studies, Zirconia-reinforced lithium silicate, fracture resistance, cementation

## Abstract

In recent years, Zirconia-reinforced Lithium Silicate ceramic (ZLS), combining lithium-silicate and zirconia features, has shown to have excellent mechanical and aesthetic characteristics. Thus, the aim of this study was to compare the fracture strength of ZLS single crowns cemented with two different cementation techniques. Twenty crowns were realised and cemented on teeth replicas achieved from an extracted premolar human tooth. The samples were divided into two groups of 10 specimens each, Glass-ionomeric cement (GIC) group and Self-Adhesive Resin Cement (ARC) group. The mechanical test was performed using a universal testing machine. The specimens were then evaluated with a scanning electron microscope (SEM) to identify for all crowns and related abutments the pattern of fracture after the breaking point. The data obtained were statistically analysed. The mean fracture toughness values and standard deviations (±SD) were 2227 ± 382 N and 3712 ± 319 N respectively for GIC and ARC groups. In fact, *t*-test showed a statistically significant difference between the two groups (*p* < 0.001). Moreover, the SEM results demonstrated portions of abutments still attached to the crown fragments in the ARC group, whilst these were not present in the GIC group. Within the limitations of this study, these results suggest the use of adhesive cementation for ZLS crowns, which significantly increase the compressive strength of ZLS restorations compared to GIC.

## 1. Introduction

Although traditional methods and metal ceramic restorations used for many years have been an acceptable combination of both mechanical and aesthetic characteristics and proven long term of follow-up [1,2], new aesthetic materials have been made available over time, showing increased aesthetic and mechanical properties [3,4,5]. Two families of dental ceramic materials have been developed on the market to date, such as: glass ceramics and polycrystalline ones. The first family is represented by feldspathic ceramics reinforced with leucite or lithium disilicate or reinforced lithium silicate. Among these, lithium disilicate represents a good compromise in terms of optical and mechanical characteristics. Specifically, it is composed of 65 vol% lithium disilicate, small needle-shaped crystals (3–6 μm × 0.8 μm) inserted in a glass matrix, with a 1 vol% porosity [6,7], showing advantageous mechanical characteristics (flexural strength: 350 MPa; fracture toughness (KIC): 3.3 MPa∙m^1/2^; heat extrusion temperature: 920 °C and thermal expansion coefficient (CTE): 10.6 + 0.25 ppm/°C) [8]. The glass ceramics, due to their inherent translucent characteristics and high optical properties, actually represent a valid alternative to the polycrystalline ceramic materials [3]. Despite this, in relation to mechanical characteristics, glass ceramics have basic limitations which discourage their use in some evaluated clinical situations. Specifically, during the years, some authors showed that lithium disilicate could be used for single anterior and posterior crowns and small bridges (up to 3 units) [3,9]. In addition, others demonstrated a low mechanical ability to restore posterior teeth with such a material [3]. 

On the other hand, the second family is represented by polycrystalline ceramics, which is composed mainly of zirconia in its various forms: alumina, stabilised zirconia and zirconia toughened alumina [8,10]. Among polycrystalline materials, the stabilised zirconia (ZrO_2_) demonstrates excellent mechanical properties that can be used for monolithic restorations for both crowns and bridges. In fact, zirconia is characterised by favourable mechanical properties (toughness: 5–10 MPa∙m^1/2^, flexural strength: 500–1200 MPa, Young’s modulus: 210 GPa) and acceptable optical characteristics [11,12,13,14]. Contrarily, monolithic zirconia restorations can suffer aging process even after short working periods; however, such a phenomenon seems to not affect its mechanical properties [15]. 

In addition, one of the main differences between the two families is represented by the different types of cementation to which they can be subjected. Undoubtedly, the vitreous ceramics guarantee satisfactory characteristics when an adhesive cementation is performed [16].

On the other hand, for conventional glass ceramic restorations, the adhesive technique is crucial to have a high quality and lasting bond. With the meaning to establish the most successful clinical protocols and its drawbacks, these techniques have been analysed and studied for years [17,18]. In fact, a variety of cementation techniques have been applied to modern integrated ceramics. Zinc oxyphosphate, zinc polycarboxylate and traditional glass ionomer cements harden by an acid-base reaction, hence the tendency to exacerbate surface irregularities in ceramic restorations [19]. Moreover, glass ionomer cements are subject to premature degradation and imbibition of water, resulting in microcracks and eventually to cement fractures [20]. Meanwhile, the resin-modified glass ionomer cements harden through a combination of auto or light curing polymerisation and acid-based reaction. The combination of advantages of the chemical adhesion of traditional glass ionomer cements, with the advantages of the composite resin, leads to an improvement in the toughness, fracture and wear resistance [21]. Thus, the glass ceramics restorations should be cemented with the use of resin-based cements by adhesive techniques which increase their fracture resistance and, consequently, ensure better long-term performance [22,23,24,25,26]. Considering the fragility and the limited flexural strength of glass ceramics, the use of cements based on composite resin indeed allows an increase in the fracture resistance of the restoration and improvement in its duration. In fact, comparing the resin-based cements with the traditional cements (such as the polycarboxylate cements of Zn, Zn phosphate or GIC), the former has higher values of compression strength and, as a result, they ensure a better support to ceramic restorations [27,28].

On the other hand, due to its polycrystalline chemical structure, zirconia cannot be subject to common surface acidification methods with the use of hydrofluoric acid; therefore, the cementation necessarily has to rely on mechanical/chemical surface treatments, possibly followed by application of silane [27].

Zirconia is usually considered a metal-free material but, from a physical-chemical point of view, it is a metal oxide with ceramic characteristics [8]. For these reasons, the presence of retentive preparations for full coverage restorations, and the use of water-based luting agents or hybrid cements is necessary to ensure the zirconia crowns retention [29,30,31].

The continuous search for ideal restorations materials has led over the years to the introduction of a promising new material. Recently released onto the market has been the zirconia-reinforced lithium silicate ceramic (ZLS), a vitreous lithium-silicate matrix with approximately 10% by weight of zirconia dioxide. This kind of ceramic material should combine the high mechanical properties and an easy intraoral polishing of zirconia with the high translucency optical characteristics typical of vitreous ceramics [10].

Manufacturers recommend the use of adhesive agents for the cementation of ZLS restorations, however, the possibility of using conventional glass ionomer-based cements (GIC) is also foreseen.

Thus, the aim of this study was to compare the two different cementation techniques, GIC vs. Adhesive Resin Cement (ARC) of monolithic ZLS crowns, in terms of fracture strength. The null hypothesis was that there were no differences between the two types of cements used of the ZLS crowns’ fracture strength.

## 2. Materials and Methods

### 2.1. Study Design

The present in vitro study evaluated the fracture strength of ZLS single crowns after the use of two different methods of cementation. A total of 20 replica teeth were performed on, starting from a single, medium-sized upper first premolar, extracted for periodontal reasons at the dental clinic of the Department of Medical, Oral and Biotechnological Sciences, University of Chieti-Pescara, Chieti, Italy. The patient signed a written informed consent to the use of the tooth after its extraction for the abovementioned purpose. 

According to Kashkari et al. [32], a sample size of 10 specimens per group was calculated to have a minimum difference between the two groups in terms of fracture toughness. The value of *α* was determined at 0.05 while the power of the test was 0.80. For the calculation, the Pass 3 software was used and specifically the Two-Sample T-Tests taking Equal Variance. Moreover, in the same study, 18 samples were used in three different groups. Thus, the sample size of 20 is sufficient to have a statistically significant difference in cases between the two groups analysed [32].

### 2.2. Sample Preparation

**Figure 1:**
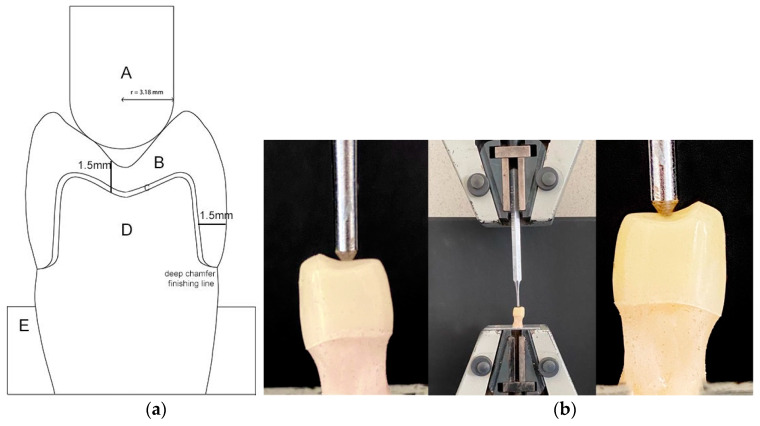
(**a**) Graphical representation of sample preparation: (**A**) tungsten carbide pilot punch with a radius of 3.18 mm; (**B**) Zls crown; (**C**) cement; (**D**) tooth abutment replica of resin composite; (**E**) specimen holder of methacrylic resin; (**b**) an explanatory image of the samples during the test.

A medium-sized upper first premolar (n = 1) was chosen as a master element and adequately prepared to receive a ZLS prosthetic crown. After the extraction, a 1.5mm axial and occlusal anatomical reduction, with a preparation angle of 4° both cuspidal sides preserved and a deep chamfer finishing line with rounded internal corners was carried out, as shown in Figure 1. A high-speed handpiece with a diamond bur (medium grit) under water irrigation, at 300,000 revolutions/min (25 CHC, KaVo Dental GmbH, Biberach, Germany), was used to ensure consistent torque and grinding speed during the preparation. To ensure complete correspondence of the preparation to the indicated guidelines, a cutting template was used as a guide. Subsequently, the impression of the prepared element was taken with the use of a putty-light biphasic polyvinylsiloxane impression material (Virtual; Ivoclar Vivadent, Schaan, Liechtenstein) as previously described [33]. Moreover, the (n = 20) replicas of the reference master element were obtained following the methodology of Coelho et al. [34]. Then, the impressions were filled with 2.0 mm of each progressive layer of composite resin (Tetric EvoCeram Bulk Fill, Ivoclar Vivadent, Schaan, Liechtenstein) under an optical microscope at 20× magnification (OPMI Movena, Carl Zeiss, Oberkochen, Germany) to ensure the absence of bubbles. Subsequently, the composite resin was polymerised following the manufacturer’s instructions. Afterwards, a digital impression was acquired through a 3D scanner (inEos Blue; Sirona Dental GmbH, Walsbeu Salzburg, Austria). Using the available data, an anatomically correct crown was Computer Aided Designed for the master element, which was subsequently milled using a four-axis CAM system (inLab MXXL, Sirona Dental GmbH, Walsbeu Salzburg, Austria) starting from non-crystallised blocks of ZLS ceramic (Suprinity LS-14 LT A3, Vita Zahnfabrik, Bad Sackingen, Germany). After the milling procedure, the twenty crowns were finally subjected to a thermal crystallisation process with simultaneous glazing (Plus Glaze LI Spray, Vita Zahnfabrik Bad Säckingen, Germany) following the manufacturer’s instructions.

### 2.3. Crown Cementation

Before cementation, the internal fit of each individual crown on the composite resin replicas was checked with the use of a black fluid silicone (Fit Checker Black, GC Europe, Leuven, Belgium) under an optical microscope at 20× magnification. Then, any friction areas were removed with the use of fine-grained diamond burs. The 20 crowns were divided into two groups and the cementation was performed as follows:

In Group GIC (n = 10), it was performed with (Ketac Cem, 3M ESPE, Seefeld, Germany) powder/liquid, mixed according to the manufacturer’s recommendations.

In Group ARC (n = 10), it was performed firstly by etching the crowns with 4.9% hydrofluoric acid (Vita Ceramics Etch, Vita Zahnfabrik Bad Sackingen, Germany) for 20 s; subsequently, the acid was removed with the use of an air/water spray syringe and any residues were eliminated with an ultrasonic bath in 98% alcohol for 3 minutes. After drying, a one-component silane (Monobond S, Ivoclar Vivadent, Schaan, Liechtenstein) was applied for one minute. Then, the application of the adhesive (Scotchbond Universal Adhesive, 3M ESPE Saint Paul, MN, USA) was carried out without polymerizing the contact surface between crown and tooth. Finally, the dual cement (Relyx Ultimate, 3M ESPE Seefeld, Germany) was applied inside the crowns, which were positioned on the replicas of the master dental element. All excesses of cement were eliminated and a curing procedure for 40 seconds on each surface was performed. The cementation of both groups was carried out under a constant static load of 50N, as previously described [35]. Subsequently, before proceeding with the experiment, the samples were left in distilled water at 37 °C for 7 d as reported. In fact, the required effect was only to simulate a dimensional variation, therefore we considered the use of distilled water sufficient for this purpose [34].

### 2.4. Mechanical Test

The sample’s holders were prepared as follows: silicone moulds (3 cm in diameter, 2 cm in height) were filled with methacrylic resin (Orthocryl, DENTAURUM GmbH & Co. KG, Inspringen, Germany); at this point the abutment tooth composite replicas were inserted perpendicularly to the resin surface of the sample’s holder, so that the most apical point of the margin preparation was placed at 2 mm from the resin surface, to ensure a repeatable positioning of the samples both intergroup and intragroup. To minimise the bias on samples preparation, silicone guides have been used. We assumed in this study that the elastic modulus of the material used in the manufacturing of standardised abutments (15.5 GPa) is included within the human dentine range (10–16 GPa) [36,37]. The test was performed using a universal testing machine (Lloyd LR30K, Lloyd Instruments, Ametek STC, Bognor Regis, UK) equipped with a 30 KN load cell. The load was applied through a tungsten carbide pilot punch with a radius of 3.18 mm applied on the central pit of the tooth in order to have a two-point contact, at the speed of 1 mm/min as shown in Figure 1. All crowns were subjected to breaking load. Following the mechanical test, the most representative samples of both groups were selected for examination under a scanning electron microscope (SEM) (EVO 50 XVP with LaB6, Carl Zeiss S, Oberkochen, Germany). Samples were prepared and analysed according to a previously described procedure [38]. Briefly, the samples were sputter coated with gold (K 550, Emitech Ltd., Ashford, Kent, UK) and stored in a sample holder. The SEM set-up was equipped with a tetra solid-state back-scattered electron detector, and was operated at 30 kV accelerating voltage, 10 mm working distance and 870 pA probe current. The images were captured with 20 scans using a line-average technique [39]. These images were stored as TIFF files before their elaboration using Image-Pro Plus version 6.0 (Media Cybernetics Inc., Bethesda, MD, USA), in order to highlight the area of fracture and the surface topography.

### 2.5. Statistical Analysis

The results are presented as mean and standard deviation (±SD). The data were analysed with descriptive statistics (Kolmogorov–Smirnov test) to evaluate whether they had a normal distribution. Student’s *t*-test for unpaired data was performed. A *p*-value < 0.05 was considered statistically significant. Statistical analysis was performed using a computerised statistical software (SPSS V. 24-0-IBM Corp., Armonk, NY, USA).

## 3. Results

**Figure 2:**
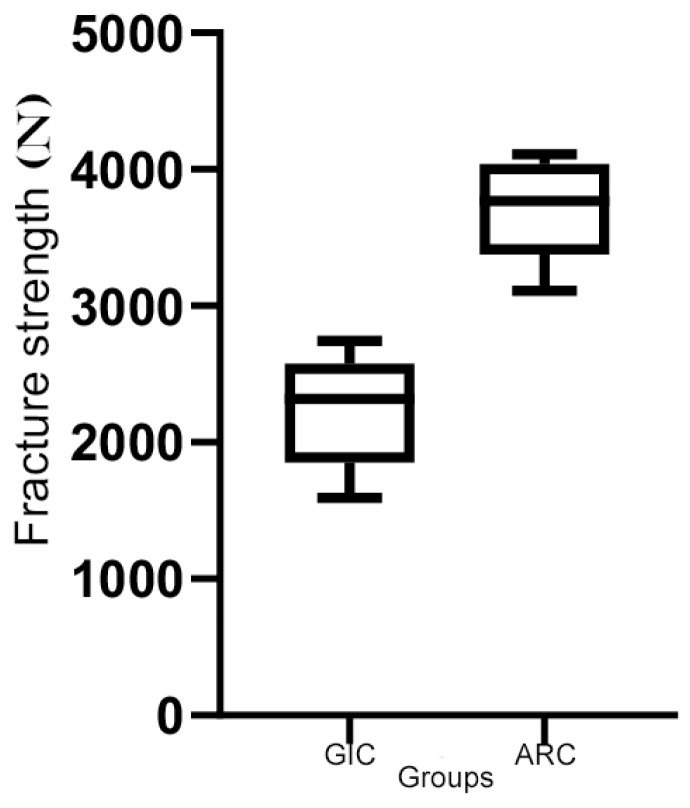
Graphical representation of fracture toughness values.

**Figure 3:**
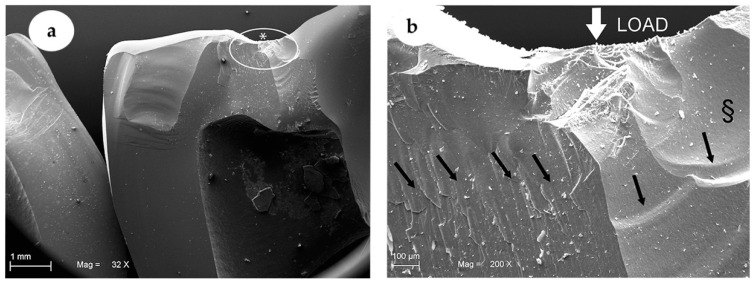
SEM images showing representative ZLS crown of GIC Group. In (**a**), (32× magnification) the tracture of a ZLS crown is shown. The * indicates the area showed at a higher magnification in (**b**) (200× magnification). Meanwhile, the white arrow shows the load application point. No residual portions of the abutments were attached to the crown. The § shows the fracture pattern and the presence of “mirror” areas near the point of force application. Black arrows indicate a fracture surface with several “hackle” areas and the direction of fracture propagation.

**Figure 4:**
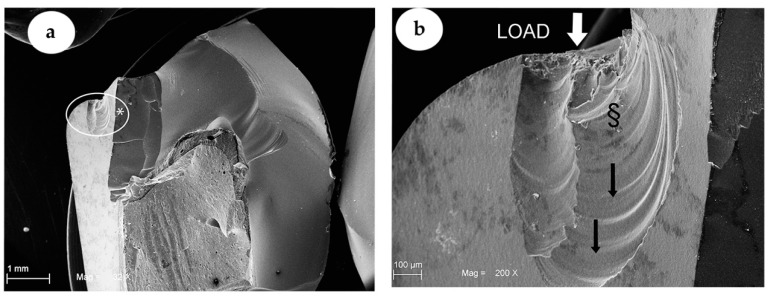
SEM images showing representative ZLS crown of ARC Group. In (**a**), (32× magnification) the fracture of ZLS crown is shown. The * indicates area showed at a higher magnification. It is possible to see the abutment attached to the crown in (**b**) (200× magnification). The white arrow shows the force application point. The § demonstrate the “mirror” areas near the point of load application. Moreover, a fracture surface with less “hackle” areas compared to GIC Group is shown, whilst the blackarrows indicated the direction of fracture propagation.

All the crowns tested reached the breaking point, sometimes causing the abutments breakings. Specifically, the mean fracture toughness values (±SD) obtained by the two groups were 2227 ± 382 N for the GIC group and 3712 ± 319 N for the ARC group, as reported in Figure 2 and Table 1. The statistical analysis demonstrated a statistically significant difference indicated with asterisk (*) between the two groups analysed, as shown in Table 1. The SEM analysis as shown in Figure 3 and Figure 4 confirmed the numerical results.

In detail, under SEM evaluation, a catastrophic failure for all crowns and related abutments was noted for both groups. Moreover, all the GIC group samples failed without residual portions of the abutments attached to the crown fragments, as shown in Figure 3. 

On the contrary, the ARC group specimens, adhesively cemented, showed portions of abutments still attached to the crown fragments (Figure 4), indicating a better adhesion of the two parts. In both groups, the fractographic evaluation on fracture surface of the ZLS material showed a “mirror” pattern area near the point of force application. In addition, the GIC group revealed a fracture surface with several “hackle” areas (Figure 3a). These results were more evident at a higher magnification, as shown in Figure 3b. Meanwhile, the ARC group specimens showed a fracture surface with “hackle” areas less representative, beyond the “mirror” area specifically shown in Figure 4a,b.

## 4. Discussion

The null hypothesis under test was rejected, demonstrating that there was a statistically significant difference in terms of fracture strength due to the type of cementation used.

Over the years, the mechanical and aesthetic characteristics of metal-free restorations have been widely evaluated. The introduction of the ZLS has made it possible to combine the excellent aesthetic performance of glass ceramics with the performance of zirconia. The marginal adaptation, translucency and yield following the aging process were assessed, positively evaluating the qualities of the material [40]. Considering the recent introduction of the ZLS material on the market, there are no long-term follow-ups studies available [41].

Despite this, some precautions during the delivery of the ZLS crowns can improve the performance of ceramic restorations. Among these, the role of the cementation technique is still controversial. Some authors have shown that the cementation technique does not affect the long-term yield of ceramic restorations [42]. On the contrary, other studies have shown how cementation can influence the follow-up or survival rate of the prosthetic restorations, as well as the fracture resistance. The differences in glass ceramics were statistically significant [40,43,44]. In this regard, the present results encourage the use of ZLS with a cemented technique, demonstrating a statistically significant increase in fracture resistance. Preis et al. in 2015 assessed the influence of cementation of CAD/CAM-fabricated ZLS molar crowns [45]. Our results showed higher fracture strength values compared to the one of Preis et al. We think that these differences may have been influenced by different factors. Firstly, the authors used natural extracted teeth as the abutment test, which can negatively influence the results. In fact, despite the standardised methods, the teeth may have undergone different preparations, as well as different elasticity of the crown abutments’ different anatomies, modifying the results. In addition, the authors declared that an adhesively cemented ZLS crown failed during the chewing test, demonstrating how variations related to the individuality of the samples used may have altered the result itself. On the other hand, the authors performed the evaluations after a cyclic loading, which we did not. In fact, this presents a limit of our study.

On the contrary, being able to standardise with composite resin replicas, the results here presented were extremely homogeneous and with a clear trend in the results. Other factors may influence the results in carrying out the tests. Specifically, the factors that can influence the reliability of the results are related to the individual variables of the extracted teeth. Thus, resin replicas are preferable to evaluate the intrinsic mechanical characteristics of the materials. Moreover, this concept has already been described in the literature [34,46]. In particular, Coelho et al. declared that a discrepancy between clinically observed failure modes and laboratory in vitro testing were noted in some cases. The aim of their study was to firstly include an anatomically correct standardised computer design to generate laboratory specimens allowing for evaluation by computer mechanical simulation of single load-to-fracture failure and mouth-motion (contact-slide-lift-off) fatigue [34,46]. 

Another key factor is related to the modulus of elasticity of the acrylic resin used as the support of the samples (specimens’ holder), which can influence the susceptibility to fracture of the restorations [47,48]. The elastic modulus of the material used in the manufacturing of standardised abutments (15.5 GPa) is included within the human dentine range (10–16 GPa) [36,37]. Moreover, another factor is related to the behaviour of the resin cement in the cementation phase. Furthermore, since the characteristics and behaviour of the composite resin during the cementation phase appear definitely similar to those of human dentin, presumably the resin–ceramic interface behaves in a similar way to the dentin–ceramic interface [49].

At the same time, it is worth highlighting the work by Bindl et al. in 2006 that reported some statistically meaningful differences between conventional cementation and the adhesive technique in reference to feldspathic ceramic crown restorations, reinforced with leucite and lithium disilicate [44]. More specifically, restorations cemented with adhesive cementation showed higher fracture strength values than the samples cemented with the use of a classic zinc phosphate cement [44]. In a previous study, Mitchell et al. demonstrated that this different behaviour is due to the lower compressive strength of conventional cement compared to cements used for the adhesive technique [50]. 

In our study, the fracture surfaces in the ARC group showed a lack of “hackle” regions. This observation demonstrated how the propagation of the fracture within the material occurred at supersonic speed. This phenomenon suggests that, in terms of energy, the material is much more adhered to the substructure and consequently, that the energy accumulating there during the load test tends to be dissipated and discharged inside the mass much more quickly than in the second study group (GIC). On the contrary, for the GIC group, several “hackle” regions were easily detected under microscopic observation. This seems to confirm the fact that in these samples, there was less energy accumulated during the load test due probably to the cement failure in compression (energy annihilation) that occurred before the fracture of the sample itself. The different ability of the two groups to withstand the load, is consistent with the numerical values obtained during the test. In addition, other studies in the literature confirm the presence of different patterns at the level of the fracture lines, in case of vitreous ceramic crowns on which conventional cementing techniques have been used, rather than adhesive cementing ones [44].

The statistically meaningful differences found between the two groups during our study suggest that adhesive cementation may be the recommended procedure for ZLS crowns.

Some limitations of the present study must be taken into the consideration. In vitro studies with cyclic loading and clinical investigations are necessary to better assess the differences between different approaches of cementation, specifically applied to this new material. Furthermore, long-term follow-up studies as well as the evaluations of crowns removed after years of use will be important investigations.

## 5. Conclusions

Both groups showed a clinically acceptable resistance to fracture loads. However, the type of cement used affects the fracture development and mode for ZLS. Moreover, the adhesive cementation seems to guarantee a significantly higher fracture strength for ZLS material.

## Figures and Tables

**Table 1:** Statistical analysis of fracture toughness values. Samples showed a statistically significant difference between the groups (*p* < 0.05).

**Unpaired T-Test with Welch’s Correction**	
*p* value	<0.0001
*p* value summary	****
Significantly different (*p* < 0.05)?	Yes
One- or two-tailed *p* value?	Two-tailed
Welch-corrected t, degree of freedom (df)	t = 8764, df = 17.96
**Amount of Difference**	
Mean of column GIC	2227
Mean of column ARC	3712
Difference between means (ARC − GIC) ± SEM	1485 ± 169.4
95% confidence interval	1129 to 1841
R squared (eta squared)	0.8105
**Data Analysed**	
Sample size, column GIC	10
Sample size, column ARC	10
